# Atypical EEG Responses to Nonverbal Emotionally Charged Stimuli in Children with ASD

**DOI:** 10.1155/2020/2807946

**Published:** 2020-10-05

**Authors:** Galina V. Portnova, Aleksandra V. Maslennikova

**Affiliations:** ^1^Institute of Higher Nervous Activity and Neurophysiology of RAS, Russia; ^2^Psychiatric Clinical Hospital No. 1 Named after O.N. Alekseev of the Moscow City Health Department, Russia

## Abstract

This study focused on auditory emotional perception in children with low-functioning autism and investigated the children's response to emotionally charged nonverbal sounds which regularly induced emotional response in typically developing (TD) peers. An EEG was conducted, and emotional reactions were assessed using analog scales and images of presented sounds with additional images during the presentation of emotional stimuli. The results showed that EEG and emotional responses to the fearful sounds were similar in TD children and children with autism spectrum disorders (ASD). Both groups of children showed an increase in peak alpha frequency and power of alpha2-band and a decrease in low-frequency bands. Sounds of crying and laughter induced an atypical EEG response in children with ASD, with no change in alpha-band's power and frequency observed in them; this was contrary to the observation in TD children. The decrease in the fractal dimension detected in children with ASD only for sounds of crying and laughter correlated with the accuracy of assessment of these stimuli.

## 1. Introduction

Emotional disability is one of the major symptoms of autism spectrum disorder (ASD) [1, 2]. Children with ASD often have specific perception deficits which manifest in difficulties of recognition and interpretation of emotionally charged stimuli [3]; these include the perception of nonverbal aspects of voice prosody and facial expressions [4, 5], difficulties in recognition of emotional and mental states [6, 7], and lack of preference for the mother's voice [8, 9] and pleasant or familiar voices [10].

A deficit of emotional perception could be also associated with impaired auditory perception [9, 11, 12]. In particular, it was found that children with ASD had a higher variability of auditory ERP components compared to typically developing (TD) children [13]. Moreover, impaired auditory perception in children with ASD occurred, regardless of impaired speech perception [14, 15]. Previous studies have shown that the presence of these disabilities in even high-functioning children with autism makes it difficult for them to identify relevant information and suppress irrelevant information from emotional stimuli [16, 17]. When interacting with other people, children with ASD rarely focused on the emotional state of another person and were unable to express their emotions correctly; therefore, their behavior is often perceived as pretentious and irrelevant to the context of the situation [18–20].

At the same time, children with ASD may learn to distinguish others' emotional states by focusing on the physical characteristics of sounds, such as timbre, pitch, or loudness [21]. In particular, despite low-functioning children with ASD showing significant difficulties with recognition and interpretation of emotionally charged stimuli [22], children with Asperger's syndrome are able to cope with tasks of recognizing emotions and facial expressions and classifying other peoples' emotions [23, 24]. We hypothesized that the impact of emotionally charged nonverbal stimuli was associated both with physical characteristics of sounds and the emotional responses of children induced by their experience or reaction to an unfamiliar situation. This could lead to the correct identification of sounds by children with ASD, while others, on the contrary, caused an untypical response associated with anxiety or hypersensitivity to the sounds. Therefore, this study is aimed at investigating the perception of nonverbal emotionally charged sounds with varying categories, emotional tone, and physical characteristics in children with ASD. We hypothesized that the findings of this study will highlight the causes of perceptual difficulties in children with ASD and can be used in the rehabilitation, correction, and social adaptation of these children.

## 2. Materials and Methods

### 2.1. Participants

This prospective controlled trial was conducted from 2016 to 2018 at the Center for Children with Autism, where children with ASD underwent an EEG. The inclusion criteria for the ASD groups included the following: an autism diagnosis that was based on the ICD-10 Criteria (F84.0) and the Child Autism Rating Scale (CARS). The Autism Diagnostic Observation Schedule (ADOS) was used for all children. The exclusion criteria were children with disorders other than autism who were on the autism spectrum and/or those taking antipsychotic drugs or receiving other medical therapy.

The ASD group included 30 children with low-functioning autism who were 5.4 ± 2.2 years old, with a mean score of 43.7 ± 6.5 on CARS, diagnosed with moderate or severe autism by the ADOS-2, and a mean score of 102.7 ± 3.4 on the nonverbal scale of Wechsler Preschool and Primary Scale of Intelligence (WPPSI). The vast majority of children in this group showed symptoms of behavior disorders; however, they did not show symptoms of tactile hypersensitivity and neither did they have to be persuaded or held during the EEG recording.

The control group comprised 32 children between the ages of four and six years (5.4 ± 2.1 years) who had no history of neurological or mental illness and were not taking antipsychotic drugs or receiving other medical therapy. TD children had a mean score of 21.7 ± 4.6 on CARS, out of ASD by ADOS-2, and a mean score of 106.1 ± 3.4 on the nonverbal scale of WPPSI.

The Ethics Committee of the Institute of Higher Nervous Activity and Neurophysiology of the Russian Academy of Science (IHNA and NPH RAS) approved the study protocol following the Code of Ethics of the World Medical Association (Declaration of Helsinki) for experiments involving humans. All parents or legally authorized representatives of the children signed a written informed consent before the study.

### 2.2. Stimuli

Eight nonverbal, emotionally charged stimuli were presented using loudspeakers: a sound of a woman scream, birds singing, a dog barking, nails scratching, coughing, crying, laughter, and pink noise. Each stimulus was randomly presented 14 times, and the interstimulus interval varied randomly from 700 to 2500 ms. The duration of the stimuli was 1420-1560 ms (1507 ± 49 ms). The physical characteristics of the stimuli are presented in Table 1.

#### 2.2.1. Stimulus Assessment

**Figure 1:**
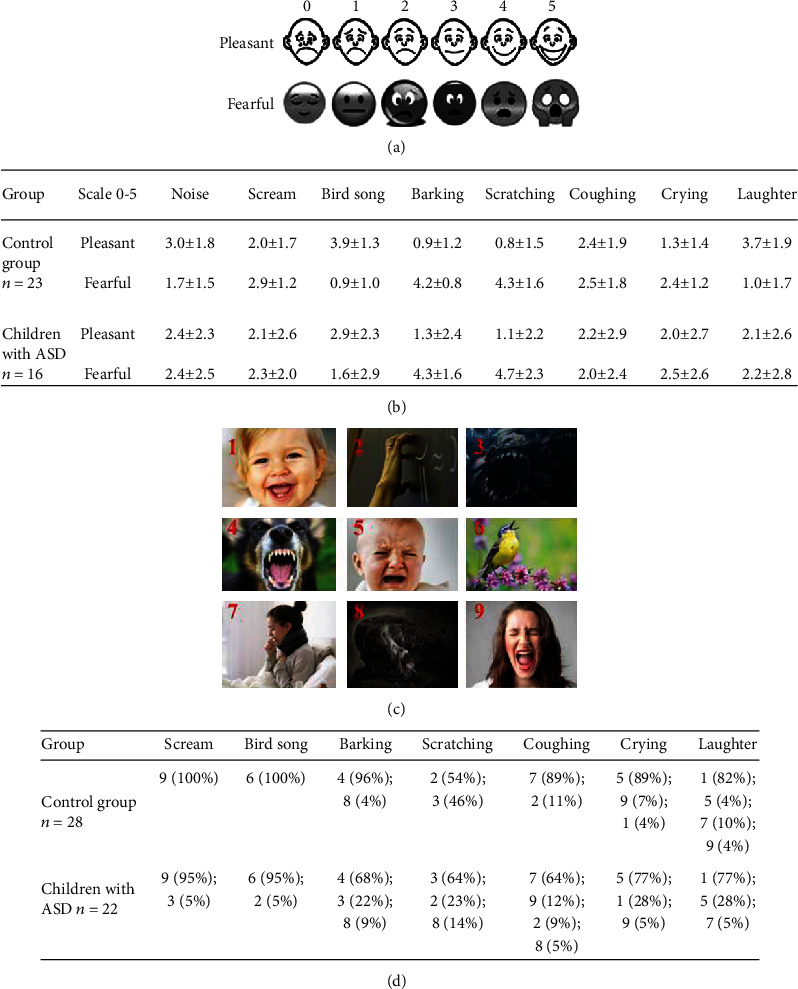
(a) Schematic example of the analog scales of pleasantness and fear (0–5). (b) Stimulus assessments using analog scales by both groups of participants. (c) Images used to identify sounds. (d) Recognition of stimuli using images in both groups.

In anticipation of the children's difficulties in understanding and following the instructions, we prepared analog scales to assess stimuli (Figure 1(a)) and instructed children to mark the desired emotion according to pleasantness (0–5) and fear (0–5). Only 23 out of 32 children in the control group and 16 out of 30 children with ASD were able to complete this task.

Children were also instructed to identify the particular sound by selecting it from one of nine pictures. In addition to seven images depicting emotional sounds, two images of monsters were included; however, the pink noise was not presented. The need to include additional fearful images was based on the hypothesis that the impaired perception of children with ASD, which was accompanied by fear and anxiety emotions, triggered a response to nonverbal stimuli. Only 28 out of 32 children in the control group and 22 out of 30 children with ASD could complete this task (Figure 1).

#### 2.2.2. EEG Registration

Resting-state EEG was assessed using a 19-channel EEG amplifier Encephalan with the recording of polygraphic channels (Poly4, Medicom MTD, Taganrog, Russian Federation) for 10 minutes. The sampling rate was 250 Hz. The amplifier bandpass filter was nominally set to 0.05–70 Hz. AgCl electrodes (Fp1, Fp2, F7, F3, Fz, F4, F8, T3, C3, Cz, C4, T4, T5, P3, Pz, P4, T6, O1, and O2) were placed according to the International 10–20 system. The electrodes placed on the left and right mastoids served as joint references under unipolar montage. The vertical EOG was recorded with AgCl cup electrodes placed 1 cm above and below the left eye, and the horizontal EOG was acquired by electrodes placed 1 cm lateral from the outer canthi of both eyes. The electrode impedances were kept below 10 k*Ω*.

#### 2.2.3. EEG Preprocessing

Continuous EEG corresponding to the stimulation and the resting state of each participant was gleaned from eye movements by an ICA-based algorithm in the EEGLAB plugin for MATLAB 7.11.0 (MathWorks Inc.). Muscle artifacts were cut out through manual data inspection. The continuous resting-state EEG of each participant was filtered with bandpass filter 0.5–30 Hz. The duration of each analyzed EEG fragment was 178 ± 22.3 s.

### 2.3. Data Analysis

#### 2.3.1. Power Spectral Density (PSD)

Fast Fourier transform was used to analyze PSD. The EEG spectrum was estimated for every 178 ± 22.3 s intervals. The resulting normalized spectra were integrated over intervals of unit width in the range of interest (2–3 Hz, 3–4 Hz,…,19–20 Hz).

#### 2.3.2. Peak Alpha Frequency (PAF)

PAF was estimated as a value of frequency with maximal PSD ranging from 8 to 13 Hz based on frequency discretization data.

#### 2.3.3. Higuchi Fractal Dimension (HFD)

Calculations were based on the examined signal bandpass filtered in the range of interest (2-20 Hz) with a Butterworth filter of order 12; IIR filter was used to compensate the phase delay and distortion using filtfilt function (MATLAB, MathWorks). HFD was evaluated using the Higuchi algorithm 24.

#### 2.3.4. Envelope Mean Frequency (EMF)

To evaluate the (de-)synchronization dynamics of the rhythms, we applied the following method. First, we calculated the envelope of the EEG signal for 2-20 Hz using Hilbert transform.

#### 2.3.5. Hjorth Complexity (HC)

HC represents the change in frequency and indicates how the shape of a signal is similar to a pure sine wave. This parameter was calculated for a wideband 1.6-30 Hz filtered signal in the following way: complexity (*y*(*t*)) = mobility(*y*′(*t*))/mobility(*y*(*t*)), where mobility (*y*(*t*)) = var(*y*′(*t*))/var(*y*(*t*)), *y*(*t*) is a signal, *y*′(*t*) is its derivative, and var(*y*(*t*)) is the variance.

### 2.4. Statistical Analysis

Linear and repeated measures ANOVAs with post hoc comparison (Bonferroni, *p* < 0.05) were used to determine group effects in EEG metrics and stimulus assessments. We analyzed a possible association between EEG metrics and the ratings of the participants' assessment of emotional stimuli and age using Spearman's correlation analysis corrected for multiple comparisons by a cluster-based permutation test using the clustering method (MATLAB toolbox for BCI) with 500 permutations at each node (the Bonferroni-corrected *p* value of 0.05). The clusters of differences for the EEG metrics were also calculated by a cluster-based permutation test using the clustering method and the Wilcoxon signed-rank test. The group differences in behavioral assessment results were calculated using the Mann–Whitney test. Only significant (*p* < 0.05) correlations and differences were reported.

## 3. Results and Discussion

### 3.1. Peak Alpha Frequency

**Figure 2:**
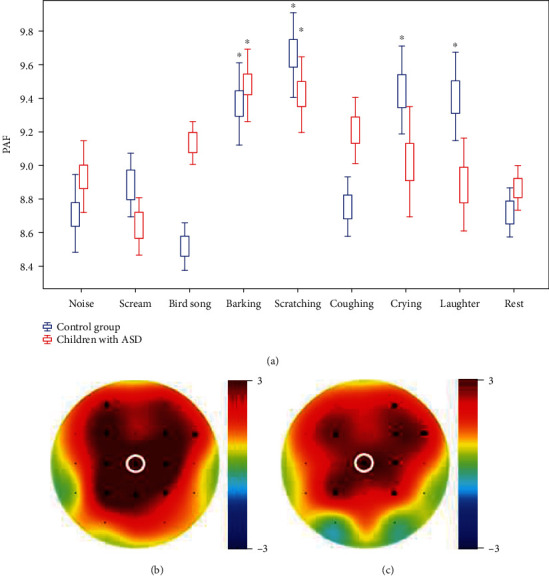
(a) Plots of PAF when listening to stimuli and rest. ^∗^Significant differences between stimuli. *x* (abscissa): the types of stimuli presented to participants; *y* (ordinate): PAF averaged for participants of each group. (b, c) The topography was depicted for sound “barking” averaged for all participants separately for each group ((b) control group, (c) children with ASD). White circle: Cz—electrode in which PAF values were depicted.

TD children showed an increase in PAF compared to the other children when listening to the sounds of barking, scratching, crying, and laughter (Figure 2). Children with ASD showed increased PAF compared to the other children only while listening to the sounds of barking and scratching (*F* (1, 60) = 7.92, *p* = 0.008).

### 3.2. Fractal Dimension (FD)

**Figure 3:**
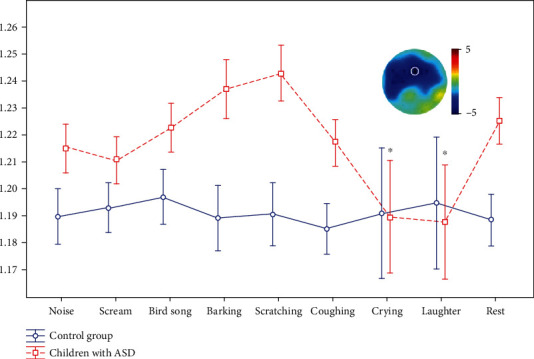
The dynamic of FD when listening to stimuli and rest. *x* (abscissa): the types of stimuli presented to participants; *y* (ordinate): FD averaged for participants of each group. ^∗^Significant differences between stimuli and rest with topography of these differences (averaged for crying and laughter) in children with ASD. White circle: Fz—electrode in which FD values were depicted.

Children with ASD had significantly higher FD compared to the control group (main group effect *F* (1, 60) = 12,923, *p* = 0.00097) for all conditions except for sounds of crying and laughter. When listening to these sounds, children with ASD showed a significant decrease in FD compared to other conditions; as a result, the values of FD were aligned between the two groups (*F*(1, 60) = 13,621, *p* = 0.0008). The ANOVA post hoc analysis revealed significant differences between the mean values of FD for sounds of crying and laughter compared to the resting state in subjects with ASD; however, there were no other significant differences. These differences were most pronounced in the frontal areas (Figure 3). Other significant differences between FD during resting states and background were not detected.

### 3.3. Envelope Mean Frequency (EMF) and Hjorth Complexity (HC)

EMF and HC were significantly higher in children with RAS compared to the TD group (the main group effect *F* (1, 60) = 17,880, *p* = 0.00008), both during resting state and stimulation (in all electrodes).

### 3.4. Power Spectral Density

**Figure 4:**
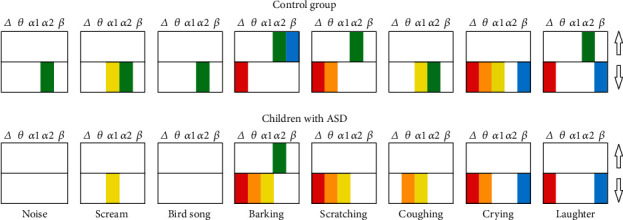
The PSD differences between stimuli and rest for delta (2-4 Hz), theta (4-8 Hz), alpha1 (8-10 Hz), alpha2 (10-12 Hz), and beta (12-20 Hz) bands. The colored columns depict significant differences which were supported by the clustering permutation test and passed Bonferroni correction. Arrows (up and down) indicate an increase and decrease compared to the background.

The PSD of the resting-state EEG showed no significant group differences. At the same time, children in the control group showed a decrease in delta- and theta-band PSD and an increase in alpha2-band while listening to the sounds of barking and scratching (which were the most fearful stimuli, according to the assessments); children with ASD showed similar EEG dynamics when listening to these sounds (the main effect *F* (1, 60) = 14,321, *p* = 0.00073; see Figure 4).

The sounds of crying and laughter, which were poorly differentiated from each other but successfully distinguished from other sounds, induced a similar EEG response. TD children showed a decrease in delta to alpha1 PSD and beta-rhythm PSD and an increase in alpha2-rhythm PSD. Children with ASD showed a similar decrease in low-frequency and beta-band PSD, but showed no significant changes in the alpha-rhythm band (group effect *F* (1, 60) = 11,875, *p* = 0.00198).

The rest stimuli induced significantly lower responses in both groups of participants accompanied by a decrease in the alpha2-rhythm's PSD in the control group and theta-to-alpha1-rhythm's PSD in children with ASD.

### 3.5. Correlation Analysis

**Figure 5:**
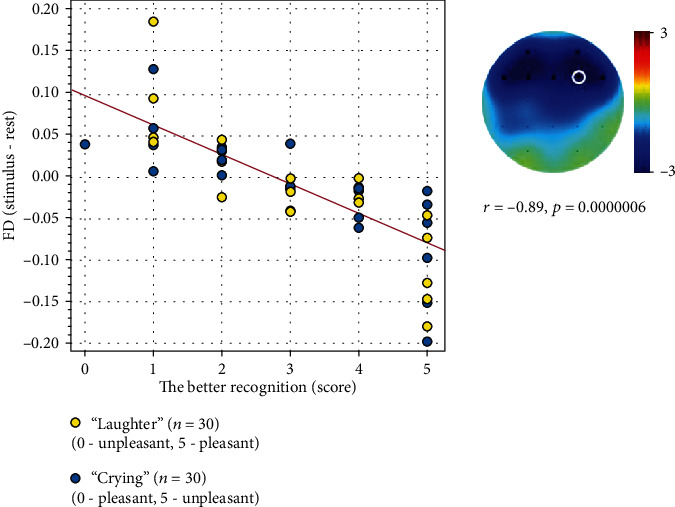
Results of the permutation test's correlation analysis between differences of FD values (the FD during rest was subtracted from crying and laughter) and assessments of these stimuli by scale (unpleasant-pleasant) unified in such a way that 0 is the incorrect recognition of the emotion, and 5 is the correct recognition of the emotion.

Pleasantness of laughter and unpleasantness of crying showed significant correlation with decrease in FD using the clustering permutation test (*r* = −0.89, *p* = 0.0000006) in frontal areas (Figure 5). Other correlations between stimulus assessments and EEG metrics did not pass the Bonferroni correction.

## 4. Discussion

Analysis of linear and nonlinear features of EEG resting states confirmed previously reported findings about EEG characteristics of children with ASD compared to TD children. In particular, we found that EMF, FD, and HC were significantly higher in children with ASD compared to the control group, whereas the PSD of the resting-state EEG showed no significant differences. These results were consistent with previous findings that showed the absence of significant differences in the resting-state EEG between TD children and children with ASD of similar age [25] and similar intellectual development [26].

Emotional perception of nonverbal sounds in children with ASD had both similarities and differences with the emotional perception of TD children. Despite the difficulties some children faced in assessing stimuli using analog scales and images, our study revealed that sounds of barking and scratching were perceived as the most fearful and unpleasant. Moreover, while the barking of a dog was frightening because of its physical features and was recognized by most children, the sound of nails scratching was frightening because of its uncertainty. For this specific stimulus, most of the children chose additional pictures with monsters. These two frightening types of stimuli showed the highest similarity in the emotional and EEG responses in both groups of children. In particular, the children in the control group showed a decrease in delta- and theta-band PSD and an increase in alpha2-bands when listening to the sounds of barking and scratching. Children with ASD showed similar EEG dynamics such as a decrease in delta- and theta-band PSD and an increase in alpha2-band while listening to these sounds. TD children and children with ASD showed an increase in PAF compared to the TD children while listening to the sounds of barking and scratching. The emotion of fear was the oldest and strongest feeling [27] and was best recognized in patients with variable mental disorders. For example, the most pronounced problems occurred when patients attempted to discriminate or express emotional states of sadness and joy, whereas in particular, patients with schizophrenia perceived and identified emotions of fear significantly easily and more accurately. These sounds caused fear with prosody of speech and fearful facial expressions [28]. However, the participants showed impairment in discrimination of happy expressions [29] and in the perception of crying sounds [30]. A similar trend was revealed for individuals with autism. As shown previously, despite some atypical responses to fearful stimuli [31], individuals with ASD showed significantly better recognition of fearful stimuli compared to other emotions. In other studies, adults with ASD showed normal ERP responses to a fearful stimulus [32].

The perception of other people's emotions, namely, the sounds of laughter and crying, showed contradictory results of emotional responses in children with ASD and TD children. The sounds of crying and laughter, which were poorly differentiated from each other in children with ASD but were successfully distinguished from other sounds, induced similar responses as the TD children: a decrease in low-frequency and beta-band PSD. At the same time, TD children showed an increase in alpha2-bands and decrease in alpha1-band PSD which were not seen in children with ASD. Moreover, TD children showed an increase in PAF compared to the resting state when listening to crying and laughter sounds, which was not found in the ASD group. Therefore, the most group differences for EEG responses to the sounds of crying and laughter were associated with alpha2-rhythm bands that increased in their frequency and amplitude in central areas. Compared with the previously obtained data, the alpha-rhythm's amplitude was associated with recognition of emotions in speech [33], while greater accuracy of the emotions' recognition was associated with higher alpha-rhythm's amplitude. The increase in PAF found in the TD group, as was shown previously, indicated variable emotional states, both positive and negative [34, 35] and could be considered a sign of cognitive activity [36]. Intention, aggression, and joy are mainly characterized by an increase in alpha-coherence, whereas a decrease is seen for anxiety and sorrow [37].

At the same time, the emotional sounds of crying and laughter induced a decrease in FD in children with ASD compared to other conditions; as a result, the values of FD became similar to the rates of the control group. As was shown previously, the FD of EEG was associated with the BOLD signal of the limbic system [38] and could be used to assess the emotional tone of presented stimuli as well as the variable emotional states of participants [39, 40] such as arousal [41] or an emotional stress state [42]. The decrease in FD in children with ASD could be associated with emotional responses to laughter and crying and also could be explained by higher attention to these stimuli. Whatever the reason for the decrease in dimension in children was, it was associated with the correct identification of the emotion of sound. In particular, despite problems in distinguishing between crying and laughter, the children with ASD who correctly identified stimuli as pleasant or unpleasant showed the highest decrease in FD. Therefore, combined with previous findings, we report that with the decrease in FD, which could be associated with variable mental and emotional states [43, 44], the better recognition of this group of stimuli indicated certain cognitive or mental activity when listening to the sounds of crying and laughter. Thus, we assumed that children with ASD exhibited an atypical response when listening to the sounds of crying and laughter, and their emotional and cognitive responses were modulated by some self and environmental factors. Finally, despite our results requiring further investigation, the application of sounds of crying and laughter could have a certain potential in the social rehabilitation of children with ASD.

## 5. Conclusions

The study revealed that the EEG and emotional responses to fearful nonverbal sounds among TD children and children with ASD were similar and were accompanied by increases in PAF and power of alpha2-bands and a decrease in low-frequency bands. Sounds of crying and laughter induced atypical responses in children with ASD resulting in a lack of change in alpha-rhythm's PSD and frequency, typically found in TD children. The decrease in FD, compared to the resting state and other stimulation, which was found in the ASD group, elicited typical FD rates in the ASD groups. Overall, the findings demonstrated that the nonverbal sounds could be potentially used in the diagnosis of children with ASD as a marker of their emotional deficit. The findings can also form the basis for the development of impact rehabilitation programs for children with low-functioning autism.

## Figures and Tables

**Table 1:** Physical parameters of auditory stimuli.

	Loudness (RMS power (dB))			Pitch (Hz)			*L*∗*P* variability
	Mean	St. D.	Variability	Mean	St. D.	Variability	
Noise	21.03	0.46	0.02	1417.8	239.20	0.17	0.00
Scream	38.85	2.33	0.06	1491.6	394.02	0.26	0.02
Bird song	25.82	6.32	0.24	2369.0	496.17	0.21	0.05
Barking	34.22	13.12	0.38	1190.1	618.36	0.52	0.20
Scratching	33.95	2.84	0.08	1949.7	202.93	0.10	0.01
Coughing	32.45	7.32	0.23	1380.2	702.74	0.51	0.11
Crying	17.26	4.63	0.27	1199.2	305.94	0.26	0.07
Laughter	35.17	3.78	0.11	1576.7	155.34	0.10	0.01

## Data Availability

The data used to support the findings of this study can be requested from the corresponding author.
